# Precise mass determination of single cell with cantilever-based microbiosensor system

**DOI:** 10.1371/journal.pone.0188388

**Published:** 2017-11-21

**Authors:** Bogdan Łabędź, Aleksandra Wańczyk, Zenon Rajfur

**Affiliations:** Institute of Physics; Faculty of Physics, Astronomy and Applied Computer Science, Jagiellonian University, Krakow, Poland; Leibniz-Institut fur Pflanzengenetik und Kulturpflanzenforschung Gatersleben, GERMANY

## Abstract

Having determined the mass of a single cell of brewer yeast *Saccharomyces cerevisiae* by means of a microcantilever-based biosensor *Cantisens CSR-801 (*Concentris, Basel, Switzerland), it was found that its dry mass is 47,65 ± 1,05 pg. Found to be crucial in this mass determination was the cell position along the length of the cantilever. Moreover, calculations including cells positions on the cantilever provide a threefold better degree of accuracy than those which assume uniform mass distribution. We have also examined the influence of storage time on the single cell mass. Our results show that after 6 months there is an increase in the average mass of a single yeast cell.

## Introduction

Mass constitutes one of the most important physical parameters of a cell. However, its regulation and involvement in cellular processes is still poorly understood. The main reason being that it is quite difficult to directly determine the mass of a single or even a few cells. There are a few established methods such as optical quantitative phase imaging (QPI) [1–3], pedestal resonant sensors [4], suspended microchannel resonator [5–7] or microcantilever-based microbiosensors [8–9] which have been successfully used to determine the mass of individual cells. Measuring single cell mass provides an opportunity to investigate the regulatory processes and behavior of a singular cell–for there is no interference between the signals from distinct cells.

The research employed a microcantilever-based system and improved data elaboration procedure to determine the average mass of a single *Saccharomyces cerevisiae* yeast cell. This is one of only a few methods that enable the direct measurement of adherent cell mass. The sample (in our case yeast cells) requires no fluorescent or radioactive marker labelling, which could affect its mass and internal cell processes [10–12]. The method measures the mass of a few cells with high precision in a non-invasive way. Furthermore, it affords real-time measurements offering the possibility of performing 8 different, independent experiments simultaneously.

The basic principle of the cantilever-based microbiosensor is that it converts molecular biological interactions into a mechanical response–bending or changing its resonant frequency–from the elastic silicon micrometer-sized cantilever. The cantilever response is optically detected and then converted into an electronic signal [13]. High sensor sensitivity and precision allow for experiments to be performed at the cellular or even molecular level making it possible to detect the cantilever bending magnitude in a single nanometer range.Measurements can be carried out in a liquid environment as well as in the air with a controlled temperature in the measuring chamber. The interaction specificity of the cantilever biosensor response can be significantly improved by means of suitable functionalization of the cantilever surface. This is accomplished by coating the cantilever surface with specific antibodies or nucleic acid fragments [14–15]. Cantilever microbiosensors are able to operate in two independent work modes: the static, where only a cantilever bending magnitude is measured, and the dynamic, where the change of cantilever resonant frequency is determined. The working principles of the cantilever biosensor modes are described below:

1. in static mode, nanometer cantilever deflection occurs as a result of the difference between the upper and lower cantilever surface stress levels caused by the external interactions of the biomolecules deposited on one of the cantilever surfaces. The magnitude of the cantilever deflection is described by Eq 1 [13]:
  $$\Delta z=\frac{3(1-\upsilon )L^{2}}{Et^{2}}\Delta \sigma$$ (1)
  Δz is the magnitude of cantilever deflection; Δσ is surface stress; *ν* and E are the Poisson ratio and the Young modulus of cantilever, respectively; L and t denote cantilever length and thickness;
2. in the dynamic mode, a load of an additional mass, *Δm*, causes a resonant frequency shift (*Δf = f*_*1*_*-f*_*0*_) from which *Δm* can be determined [16]:
  $$\Delta m=\frac{\lambda_{n}^{4}k}{4\pi^{2}}\left(\frac{1}{f_{1}^{2}}-\frac{1}{f_{2}^{2}}\right)$$ (2)

*λ*_*m*_ is a constant for the respective resonance mode; k is a spring constant; f_0_ and f_1_ are cantilever resonant frequencies before and after load deposition.

To date, in most cell mass measurements conducted using a cantilever-based biosensor it was assumed that the loaded mass was evenly distributed on the cantilever surface or that all the loaded mass was located at the tip of the cantilever [8–9]. In such a case, Eq 2 could be used for loaded mass determination. However, in a case where a few single cells are deposited in different places on the cantilever surface this assumption is insufficient for any precise cell mass calculation and it may result in the creation of underestimated or overestimated values for the loaded mass. This is because the frequency shift caused by the loaded mass strictly depends on loaded mass position along the cantilever. In a fundamental mode the response is the highest near the free end while there is no response at all close to the clamped end. Furthermore, the resonance shift not only depends on the position of the loaded mass but also on the dimensions of the cantilever—for a short cantilever the response is higher than it is for a longer cantilever due to the higher value of frequency resonance [9]. As Dohn et all. have defined in [17], the mass of a single particle can be calculated from a resonance frequency shift with known cell position as described in Eq 3:

$$m_{Y}=\frac{m_{0}}{U^{2}(z)}\left(\frac{f_{n}^{2}}{f_{n,\Delta m}^{2}}-1\right)$$ (3)

$$U_{n}(z)=A_{n}(\cos\left(k_{n}z\right)-\cosh\left(k_{n}z\right))+B_{n}(\sin\left(k_{n}z\right)-\sinh\left(k_{n}z\right))$$ (4)

m_0_ and m_γ_ are the mass of the cantilever and the mass of a single particle respectively, *z* is the distance between the loaded mass and the fixed end of the beam, A_n_. B_n_, k_n_ are constants characteristic of a specific resonance mode and cantilever length. After attaching a particle to the cantilever, resonance frequency f_n_ changes to f_n,Δm_. Function U(z) describes the time independent mode shape of the cantilever (Eq 4). This equation can be applied to multiple separated mass objects on the cantilever [18] (assuming that the mass of every cell is the same) (Eq 5). Eq 5 allows for a precise determination of the contribution of every mass object to the resultant resonant frequency shift. This represents the theoretical base of this work.

$$m_{Y}=\frac{m_{0}}{\sum U^{2}(z)}\left(\frac{f_{n}^{2}}{f_{n,\Delta m}^{2}}-1\right)$$ (5)

**Fig 1:**
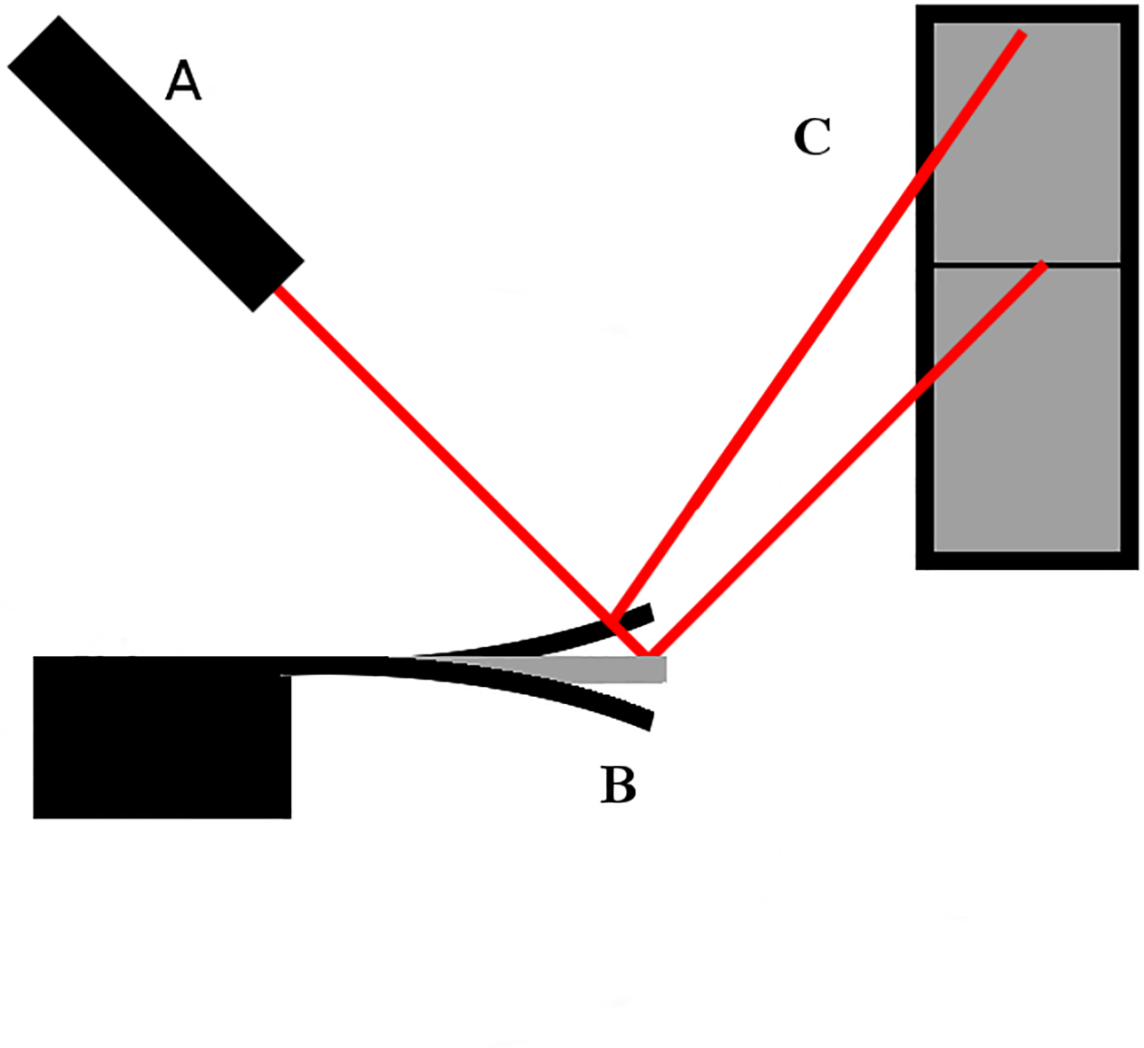
Working principle of the cantilever-based optomechanical sensor. The cantilever bending amplitude or oscillation frequency is detected by a laser-based optical system. The light from laser (A) on being deflected from the oscillating cantilever (B) falls on PSD (C) where the signal is transformed into the electronic form. Subsequently signal is sent to the computer where its magnitude is displayed on the screen.

Cantilever-based methods have been employed in many research fields. In microbiology, they were mostly used to detect the presence of microorganisms or viruses [19, 20], but also to determine biophysical parameters such as mass [8, 9] and growth rate [11–12, 21] of various biological objects. They can also be applied in studies of protein: conformational changes [22], response to ligands [23], detection of disease markers [24], specific reactions with DNA [25] and in other types of experiments. Moreover, cantilever sensors haved been used to analyze physical and chemical factors such as concentration [26], reaction heat [27], viscosity [28] or even velocity and the direction of air flow [29, 30]. Over the last few years, cantilever-based sensors have been employed in both chemical vapor detection or explosives detection [31]. Although the cantilever working principle is the same in all experiments, the deflection read-out can be monitored differently. There are optical [12, 31], piezoresistive [11] or MOS transistor-based [32] systems. This work employs an optical read-out method by Position Sensitive Detector (PSD). Fig 1 shows a diagram of the PSD cantilever-based microbiosensor.

This work focuses on the dry cell mass, which as a parameter is related to the changes in cell metabolism. Mutations or diseases can influence the number and amount of proteins produced in a cell and change their dry mass. For example, the initial mass of a cell has an impact on the onset of the DNA synthesis in the cell [33]. The ability to measure this parameter on a single cell level can provide more precise data on the molecular regulatory mechanism of this phenomenon. For example, one current work on cell growth kinetics is based on the measurements of cell volume changes. Such research should rather be based on measuring the change in cell mass instead of volume. The possibility of comparing or combining the volume and mass measurements of a single cell seems very useful in analyzing molecular processes within the cell. Nowadays, new and more accurate models of cell growth have been created and tested mostly with the help of optical methods (cell size) [34]. It seems more accurate to analyze changes in mass rather than changes in cell shape. Cantilever-based methods have a potential application in this area of research and in other areas which have not been mentioned in this article.

## Materials and methods

### Materials

Cantilever pre-cleaning was carried out with Piranha solution, prepared by mixing 95% H_2_SO_4_ and 30% H_2_0_2_ (both from Sigma Aldrich) [8].

Brewer instant yeast *Instaferm* was obtained from *Lallemand*, (Jozefow, Poland). Initially, 28 measurements were carried out with fresh yeast, while the subsequent 22 measurements were performed six months later.

Experiments were carried out on the *Cantisens® CSR 801 (*Concentris, Basel, Switzerland) cantilever-based microbiosensor, employing cantilevers type CLA-500-070-04V2 and CLA-500-070-08V. Cantilever arrays were made of silicon with a plain non-coated surface (native oxide). These types of cantilevers are designed for dynamic work mode. Each cantilever is 7 μm thick and 100 μm wide and in order to avoid any resonance between adjacent cantilevers during excitation, their lengths vary from 150 μm to 500 μm.

### Methods

We performed 50 measurements on yeast cells deposited near the free end on the cantilever surface using a microcantilever biosensor and optical microscope. Every measurement included the following steps: cantilever and chamber preparation, the first series of resonant frequency measurement, yeast cell deposition on the cantilevers, the second series of resonant frequency measuring and, finally, imaging the cantilever surfaces and data analysis.

Our measurements were performed in two time periods: 22 of them during the first term and the remainder after a half-year interval. During this period of time the dry yeast cells were kept in a fridge in an open container. In every experiment we used a chip with 6–8 cantilevers and at least 1–2 of them were without deposited yeast and acted as a negative control. Yeast deposition was controlled by comparing the images of the cantilever surfaces between every series of resonant frequency measurement. Before every experiment, a new dilution of a portion of dry yeast from the container and freshly distilled water was prepared.

### Cantilever and measurement chamber preparations

The freshly prepared Piranha solution was always used to clean the microcantilever surfaces before every experiment. Cantilever arrays were dipped for 20 minutes in the solution and, after that, rinsed three times in distilled water. Before every resonance frequency measurement the cantilevers arrays and the measurement chamber were left to dry for about 75 minutes. Before the first series of resonant frequency measurement the cantilevers chip was imaged to determine the cleanliness of the cantilever surface.

### Resonant frequency measurements

All experiments were conducted in the fundamental resonance mode of every cantilever in order to minimize the experimental uncertainty. First, cantilever oscillation frequencies were scanned to find the resonant frequencies. A full scan was performed in the frequency range from 10 to 1000 kHz with a 100 Hz step. Experimentally, the determined resonance frequencies of subsequent modes were compared with the expected, theoretically calculated values to establish the value of the fundamental mode resonance frequency. Next, the narrowed frequency range of the fundamental resonance mode on every cantilever was again scanned with a 50 Hz step. However, the final position of the resonant peak was twice re-scanned automatically by the experimental device with a step smaller than 1Hz. To determine the average resonant frequency of every cantilever, 10 such measurements were carried out at 5 or 10- minute intervals.

The lyophilized yeast were suspended in distilled water and incubated for 5 minutes at room temperature. Next, the apexes of the cantilevers were quickly immersed in a drop of freshly prepared yeast cell suspension and then immediately removed. The yeast cells adhered to the cantilever surface, whereas the amount of water that also remained on the cantilever evaporated quickly. Next, the resonant frequency was determined once again by a series of measurements performed in the way described earlier. In every experiment some cantilevers were intentionally left uncoated to act as negative controls. The second series of resonant frequency measurements for each cantilever was performed until the value of resonance frequency was stable for more than an hour. The water was evaporated from the yeast and the dry mass of the yeast cells remained in balance with the humidity in the measurement chamber. We improved the method of yeast cell deposition by changing the size of the suspension droplet before the immersion of the cantilever; consequently, the amount of water deposited on the cantilever surface was negligible. Because of this, water evaporated faster from the cells and the time needed to reach the equilibrium was shorter than before.

### Microscope images acquisition and analysis

**Fig 2:**
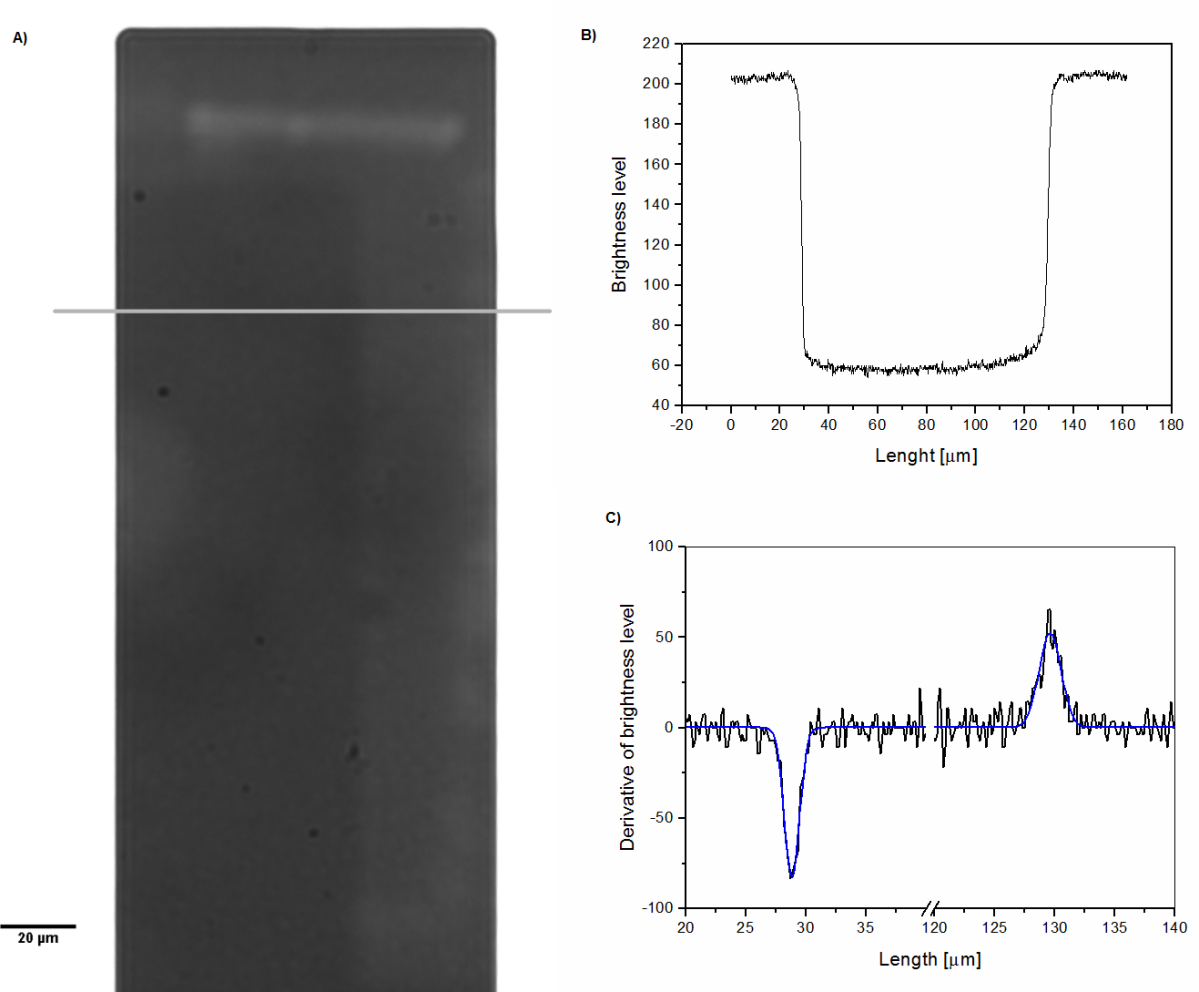
Cantilever dimensions were obtained by analyzing the line profiles on the cantilever images. A) Image of the cantilever with a superimposed line used to make the profile. B) Obtained intensity profile along a marked line. C) Derivative of intensity profile curve from Graph B. The Gauss function was fitted to the obtained peaks (line). The distance between the peak positions was interpreted as being the cantilever width. To estimate cantilever length the same procedure was performed.

**Fig 3:**
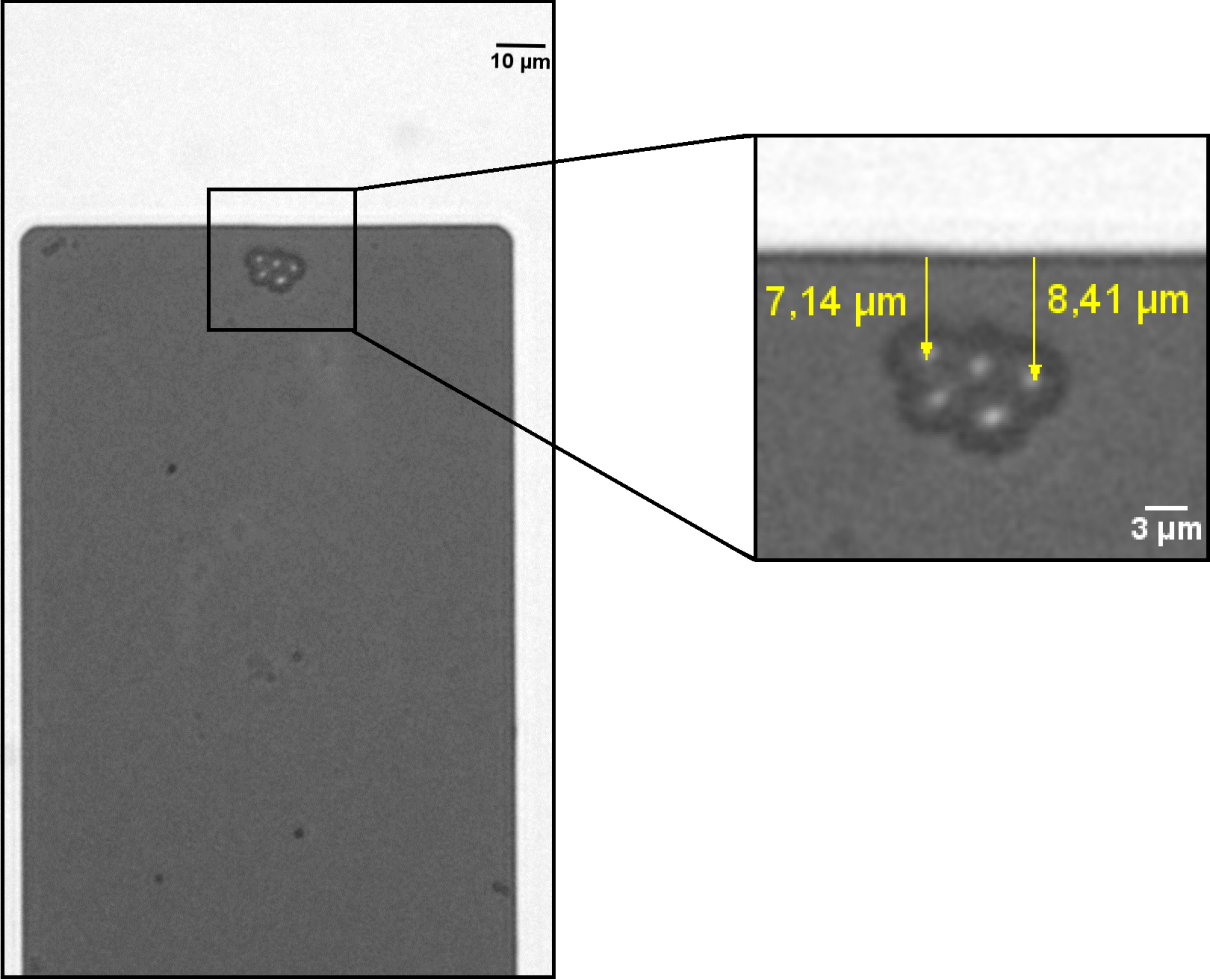
Yeast cells located on the cantilever surface. Left panel–a cluster of 5 yeast cell near the free end of the cantilever. Right panel–magnification of the square from the left panel showing the graphical determination of a cell distance from the edge of the cantilever.

A crucial step in data analysis is the determination of each cell position. Therefore, in this work, images were very carefully examined to determine the number of cells, their position and the presence of contaminations on the cantilever. Employed for this purpose was an optical microscope (Axio Observer Z1 Zeiss, Jena, Germany); the images were taken with 40x and 63x objectives (1.4 NA and 1.46 NA respectively). In order to determine the number and position of the yeast cells on the cantilever, microscope images were captured immediately after the resonant frequency measurement. First, one side of the cantilever was imaged and, after that, the cantilever array was flipped over and the other side was imaged. Due to the fact that light is absorbed in silicon, cells located on each side of the cantilever could be distinguished (through the difference in cell contrast). In some cases we were unable to count them properly because of the yeast cells that were on the opposite side of the cantilever and their overlapped images. Therefore, confocal microscopy was used (LSM 710 on Axio Observer Z1 Zeiss, Germany, Jena). This method allows for an optical sectioning not possible in wide field optical microscopy. Consequently each side of the cantilever could be separated and the position of every cell on the cantilever surface be determined with a high precision. Afterwards images were analyzed in *ImageJ (*Rasband, W.S., ImageJ, U. S. National Institutes of Health, Bethesda, Maryland, USA) to find the cantilever dimensions (Fig 2) and precise cell positions (Fig 3). On every microscope image the distance between the center of the cell and the shortest edge of the cantilever was measured and the value of the function U(z) was calculated in *OriginPro* (OriginLab Corp., Northampton, MA, USA) using Eq 4. The precise length and width of the cantilever were determined by analyzing the line profiles (Fig 2) of the perpendicular and parallel lines to the cantilever’s edges.

### Single cell mass determination

Finally, to determine single cell mass, parameters such as the number of cells and their positions obtained on the basis of image analysis, average frequency before and after the cell deposition obtained during the measurement with a microcantilever biosensor and the cantilever mass were entered into Eq 5. The mass of the cantilever was calculated from its dimensions using the silicon density of ρ_Si_ = 2300 kg/m^3^ [16].

## Results

**Fig 4:**
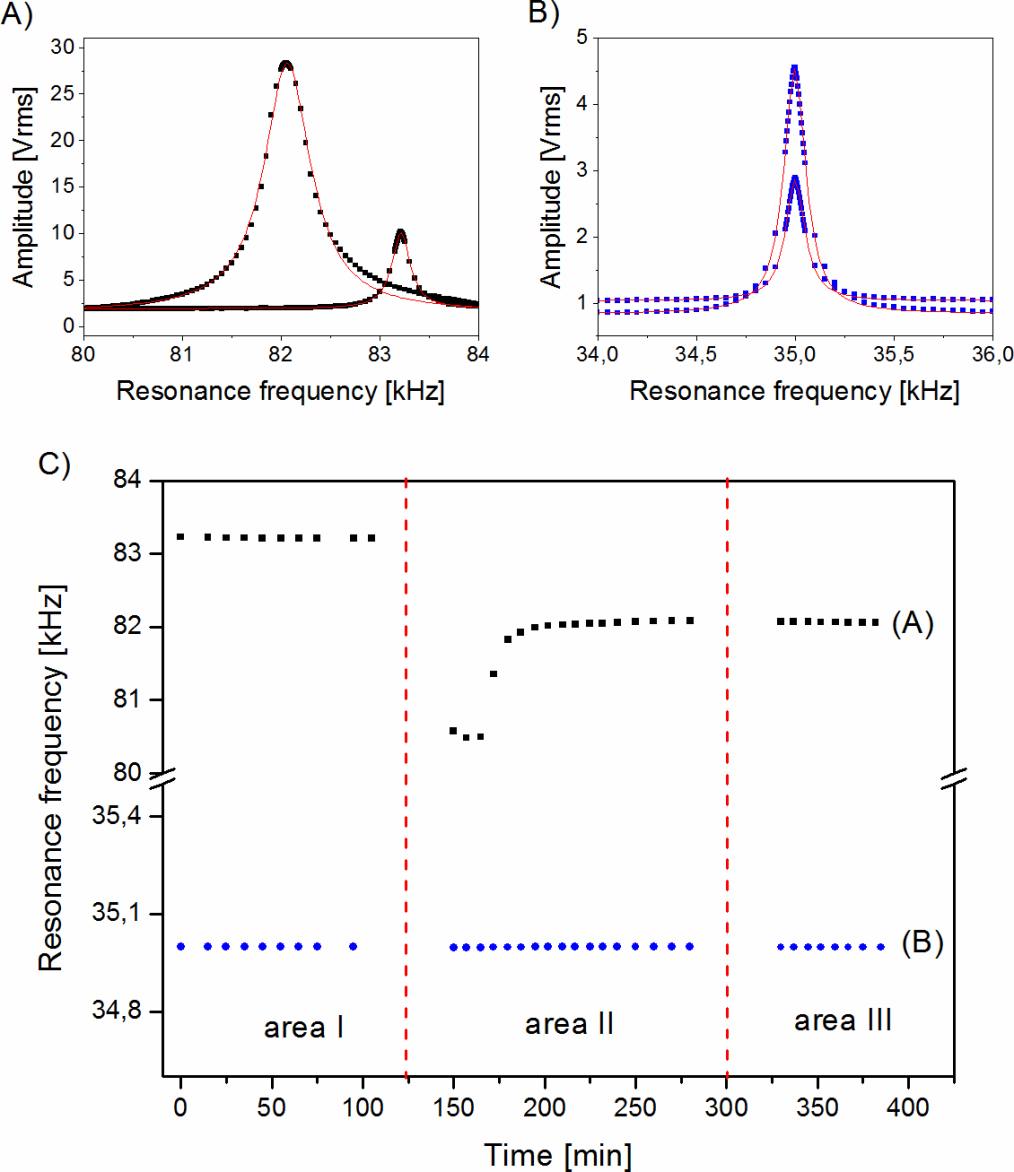
Resonant frequency shift (right peak vs. left peak) after yeast deposition (A). Reference cantilever (B) does not exhibit frequency shift. Lorentz function was fitted (lines) to experimental data (dots). The position of the peak was interpreted as the value of cantilever resonance frequency. All measured frequencies are shown on (C). Each point corresponds to a resonance frequency value at experimental time intervals. Area I shows resonant frequencies before the yeast deposition on beam A. Area II shows a transitional area stabilization of resonant frequencies (including water evaporation and cells positions shifting) for beam A. Area III shows the final stabilized resonant frequency for beam A. The resonance frequency value of the reference cantilever (B, blue dots) does not change its value during the entire measurement process.

The shift in the value of cantilever resonant frequency serves as a measure of the loaded mass. To determine precisely the value of the resonant frequency, Lorentz functions were fitted to raw data (frequency scans) (Fig 4A and 4B). The calculated position of the fitted peak was deemed to be the value of the cantilever resonant frequency. The positions of subsequent peaks were measured at equal periods of time to monitor any resonant frequency change (Fig 4C). During each measurement, the values of the resonant frequencies were very consistent, which improved the precision of this method. At least 10 resonant frequency measurements before and after yeast cells deposition were averaged in all these measurements. The values were most stable with deviations of these points in every measurement being under 3% of the frequency shift value. After the deposition of the yeast cells on the cantilevers, the value of the cantilever resonance frequency dropped as a result of its mass increase. In some initial experiments, after the deposition of yeast cells on the cantilevers, the value of the resonance frequency initially dropped and subsequently increased slightly (Fig 4C). This phenomenon was interpreted to be the result of the evaporation of the remaining water and a possible minimal shift in the cell positions, one brought about by this evaporation. With improvements in the cell loading procedure and ensuring that only a small volume of cell suspension was deposited on the cantilevers, no repeat in this phenomenon was observed.

To determine yeast cells positions, ImageJ software was used. First, we evaluated the cleanliness of the cantilever by analyzing the images taken before the experiment. We also used these images to calculate accurate dimensions of the cantilevers. Similarly, we took images after yeast deposition (before resonant frequency measurement) as well as after measurement. Then we compared these two sets of images for each cantilever to check if the position of the yeast cells had changed during the experiment. We did not observe any displacement of cells or shake losses in any of the performed experiments. The yeast cells were located near the free end of the cantilever. During water evaporation and after yeast deposition, they were pulled in clusters as a result of the water surface tension and remained in this configuration until the end of the experiment. There were no yeast cells on the cantilever’s sides because the cantilever thickness was too small in comparison to the cell size. The cell position was determined as the distance from the center of the cell to the free end of the cantilever edge It was then used to calculate the position *z* in the single cell yeast mass in Eq 4 for every cell.

**Fig 5:**
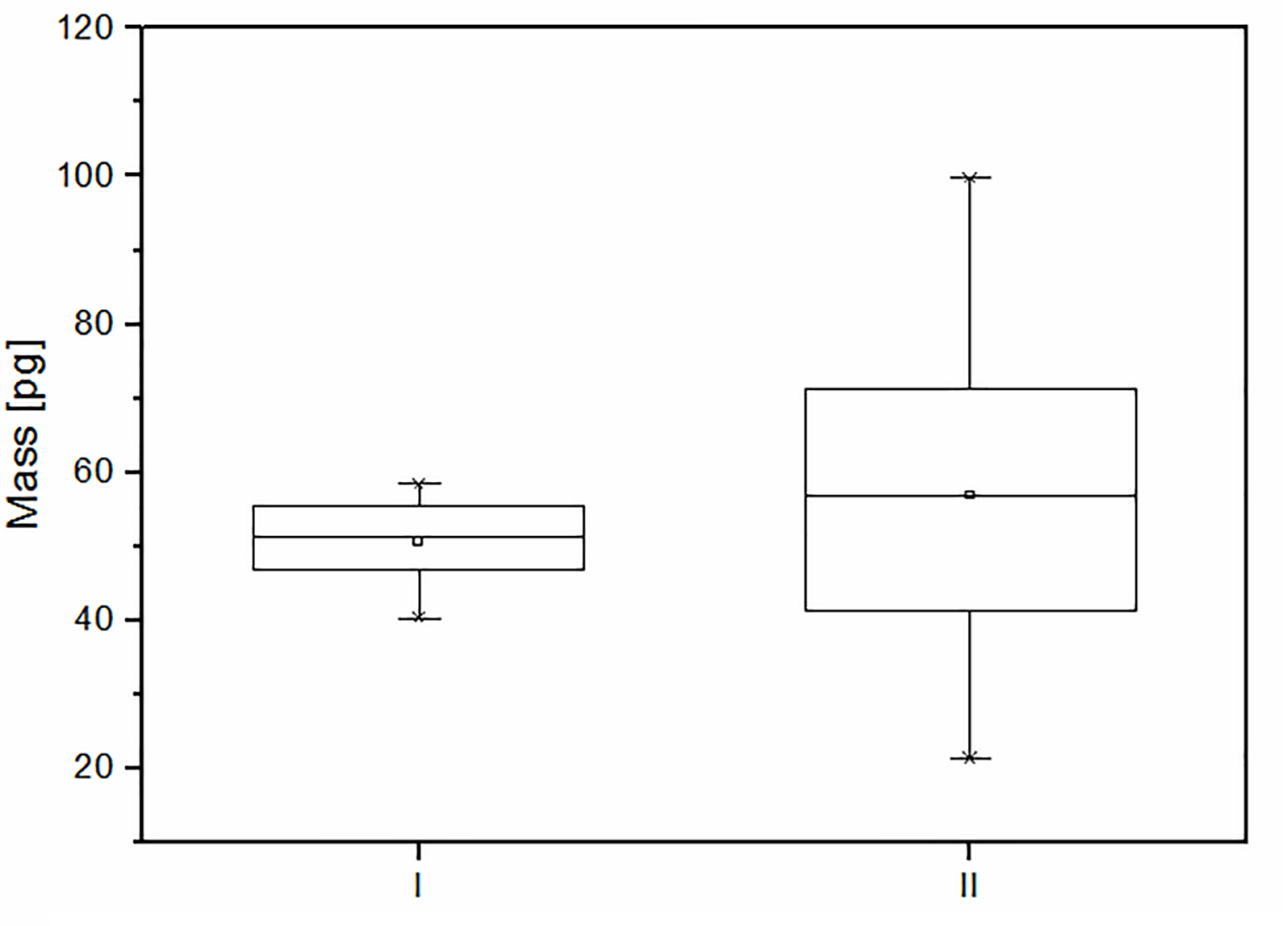
Comparison of results with and without taking into account an individual cell position along the cantilever. Box charts for an average single cell mass determination. (I)—cells positions are taken into account and (II)—assuming uniform cell distribution. 25-50-75 percent of results are marked by boxes, the minimum and maximum values are marked by whiskers. The small squares show the average value of each series.

As was mentioned in the Introduction, previous research into cell mass with cantilever-based microbiosensors, had assumed that the beam is end-loaded or uniformly loaded [9, 11]. In this work we have shown that taking the cell position into account is crucial for the precise determination of the cell mass. We have demonstrated what happens when an average cell mass is calculated in two ways: an approach including cell position (determining every cell position on the cantilever) and an approach assuming a uniform cell arrangement on the cantilever. The results of both approaches are presented on Fig 5. The mean single cell mass when every cell position was determined and included in the calculations is 47.65 ± 1.05 pg, but when uniform cell distribution was assumed, that value was 40.79 ± 2.77 pg. Moreover, taking the position of loaded masses into account gives much more precise final values than employing the other method.

**Fig 6:**
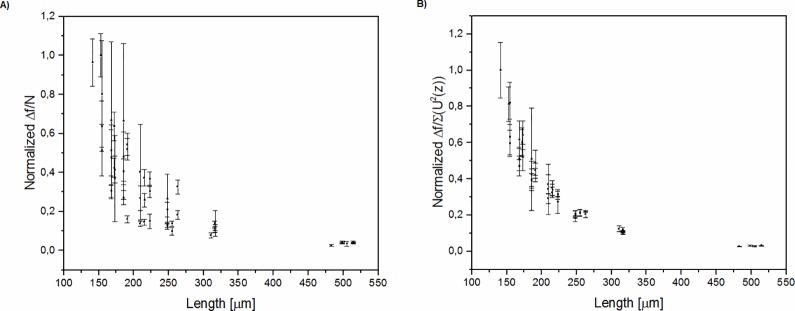
The normalized frequency response of the cantilever as a function of cantilever length. A) assuming uniform cell distribution (or the whole deposited mass on the tip of the cantilever). B) position of every deposited cell taken into consideration. In part A, every average frequency shift per deposited cell was normalized to the maximum value obtained in all experiments. In part B, the contribution of each cell to the frequency shift was taken into account by function U(z).

We also compared the cantilever frequency shift normalized by the number of cells (Fig 6A), assuming a uniform distribution of the deposited cell mass or taking into considerations the position of every individual deposited cell along the cantilever length by employing the sum of U(z) functions (Fig 6B). It has been shown that for longer beams the resonance frequency shift is smaller than for the shortest one, as was expected on the basis of theoretical assumptions [9]. However, the precision of frequency shift determination for a single cell is better for longer cantilevers. It seems that it is up to the person conducting the experiment to decide which cantilever length should be employed for particular measurements. The data also show that taking into consideration the positions of individual cells significantly decreases the data deviation and approximates it to theoretical assumptions.

**Fig 7:**
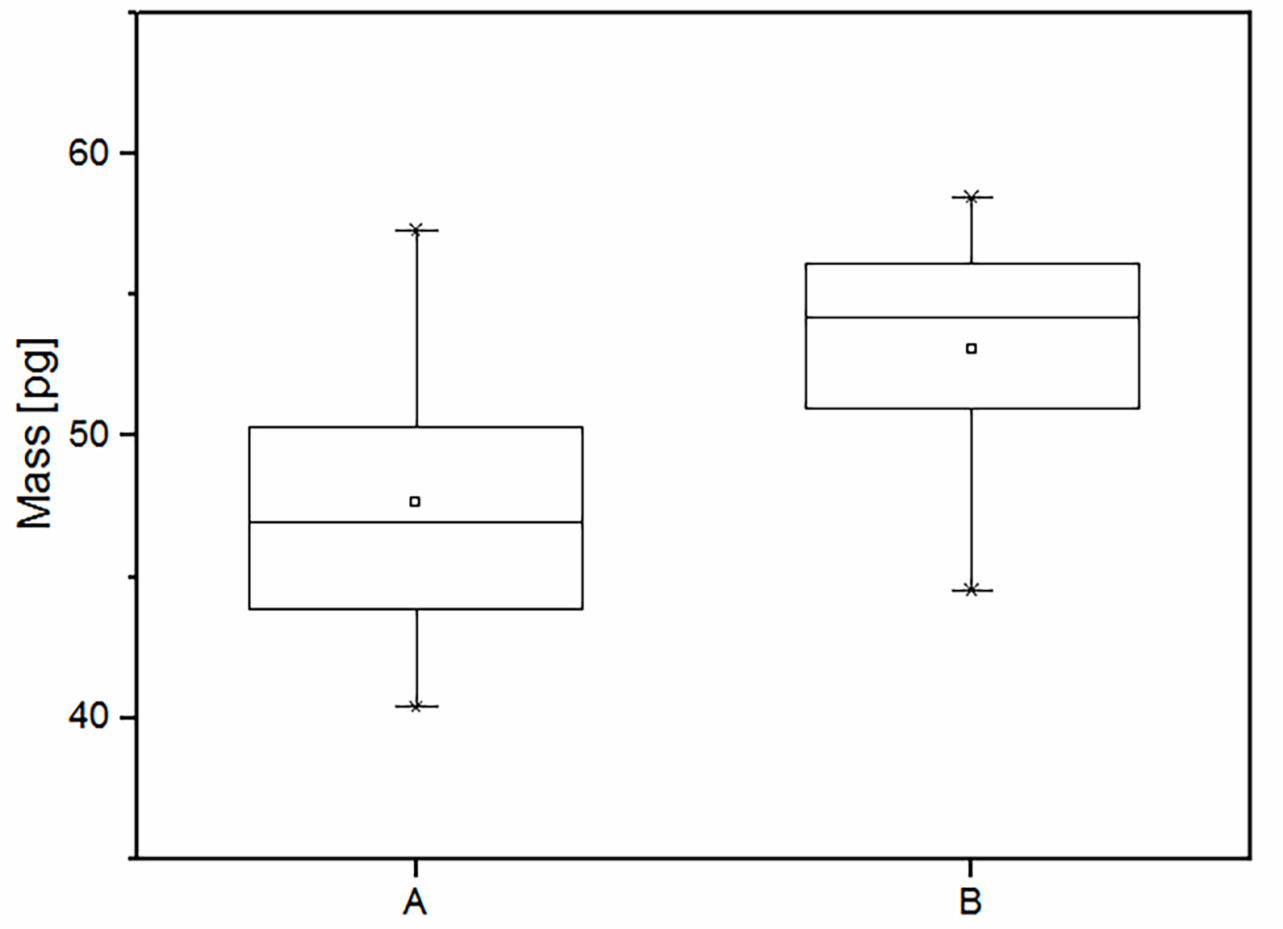
Change in the lyophilized yeast cell mass after a 6-month period of refrigerated storage. A) Initial yeast cell mass value and B) after 6 months of storage.

Next, the way the storage time of a yeast sample influences single cell mass was examined. We compared the aforementioned 22 measurements (1^st^ series) with 28 measurements (2^nd^ series) performed on the yeast sample stored for 6 months after the first experiments. The final results are shown in Fig 7. There was an increase in the mean value of the mass of a single yeast cell although the data ranges overlap. We noted that the mean single cell mass for 1^st^ series was 47.65 ± 1.05 pg (Fig 7, part A), while the single cell mass for the 2^nd^ series was 53.10± 0.73 pg (Fig 7 part B).

**Fig 8:**
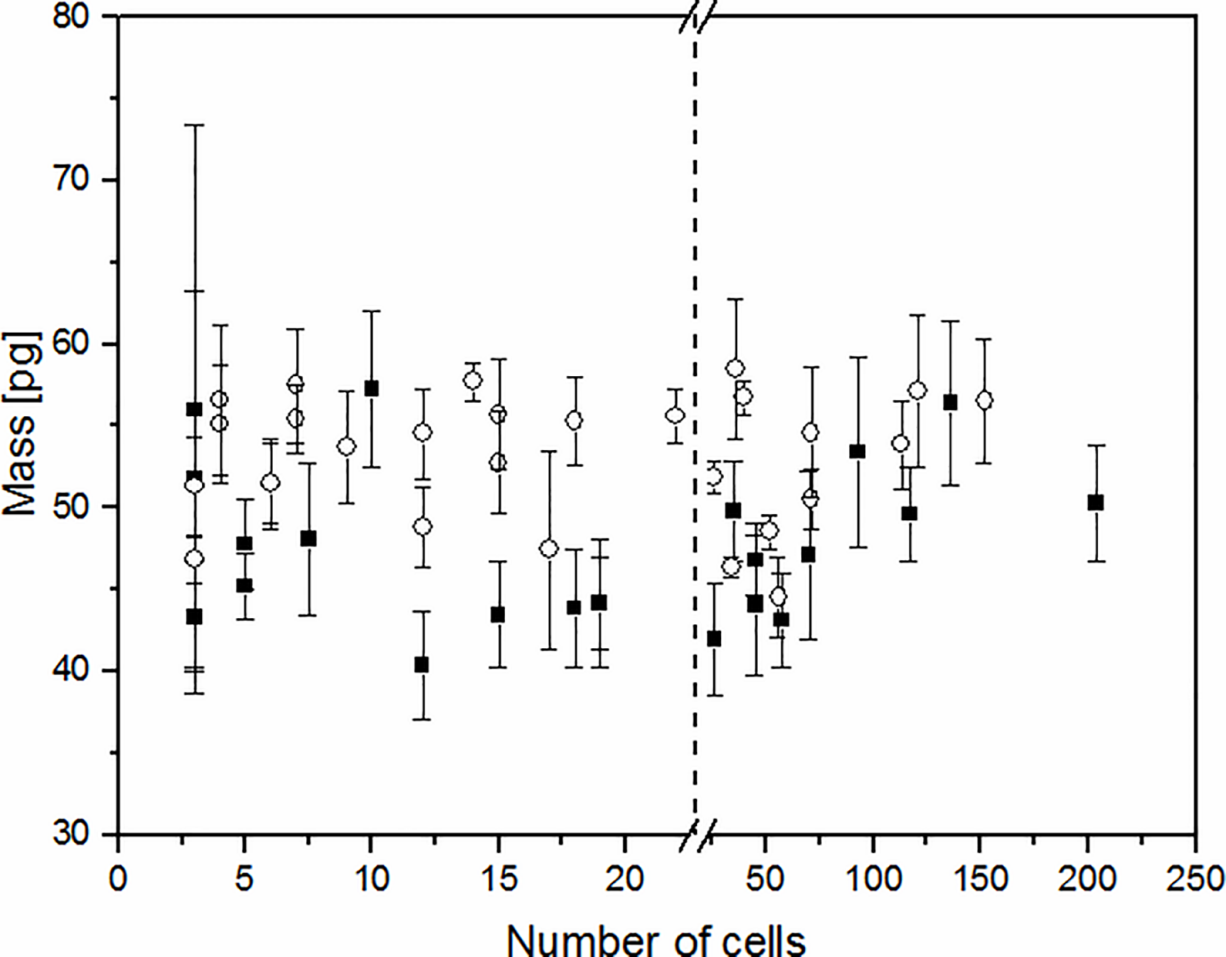
Dependence of the average single cell mass on the number of cells deposited on the cantilever. Initial series (black) and after a 6- month period of storage (white). The dashed line separates areas with a different scale.

The data shown in Fig 8 illustrate that the number of deposited cells has no influence on the measured average cell mass. However, it can be noted that for a higher number of yeast cells deposited on the cantilever the value of the single cell mass seems to be slightly higher than for a smaller number of cells. We suspect that this may be caused by residual water held in cell gaps when in clusters or simply by a miscounting of the number of cells in large cell clusters.

## Discussion

Many cellular phenomena are connected with cell mass changes. Most are also related to cell volume changes. To directly determine such links it would follow to measure the mass and volume of individual or a few cells in the physiological conditions relevant for such cells. Usually it was possible to estimate the mass of a single cell by averaging the mass of many (thousands or millions) cells, which resulted in high experimental uncertainty. Another approach is to calculate the cell mass on the basis of the optical density and volume of the cell. However, the density of the cell is not uniform while cell size depends considerably on environmental factors (such as the osmotic effect). In contrast, dry mass is an independent parameter that describes the amount of proteins and other materials inside the cell.

Different water amounts in a cell can significantly influence the results of the mass measurement. To avoid this effect, we always prepared a fresh suspension of lyophilized yeast in distilled water immediately before the yeast deposition on the cantilevers. Consequently environmental moisture has no effect on yeast mass measurements. The yeast cell wall maintains the shape and protects the cell against the osmotic effect. This was shown in [35] where the *Saccharomyces Cerevisiae* were prepared in the same way and where their size and shape did not change before and after hydration.

In this work the mass of a single brewer yeast cell was determined by means of cantilever-based biosensors systems. In our calculations we included the cells position on the cantilever and the dimensions of the cantilever, which resulted in a 3-fold improvement in accuracy than when uniform mass distribution was assumed. We employed the physical model of a vibrating cantilever, which did not take into account the stiffness of the deposited matter. The first work that described a positive frequency shift due to this effect was published more than a decade ago by the J. Tamayo group [36]. This also demonstrated a potential in the estimation of the Young modulus of adsorbed matter [37]. However, in our research, we used conditions that greatly minimize the influence of deposited material stiffness on the cantilever frequency change. First, this effect is significant when deposited matter forms layers on the cantilever surface [38]. It is also common knowledge that the stiffness effect is a major contributor when the deposited matter is located near the clamped end of the cantilever and that it decreases with the distance from the clamped end of the cantilever. Because of that the overestimation of deposited mass was observed for point masses, when they were randomly placed on the cantilever surface. In our research, we placed the yeast cells only near the free end of the cantilever, which resulted in a strong mass response and a very weak stiffness response. In [38] it was shown that the stiffness effect response is greater for an elastic (low Young modulus) adsorbent or cantilever. Moreover, the dependence of the mass reading ratio on the stiffness was calculated—the mass reading ratio is close to 1 when the adsorbent stiffness exceeds 100 kPa. In our research we used a silicon cantilever and yeast cells. Because the yeast cell wall Young modulus is close to 0.74 MPa [39] together with other conditions mentioned earlier, the stiffness effect in our research is negligible.

The single yeast cell dry mass, as determined in this work, is 47.65 ± 1.05 pg. However, the single cell mass varied from 40 pg to up to 67 pg, though most of the results are in the range 44 to 51. This shows that changes in the mass of individual cells can be correlated with the biological processes taking place in these cells. This opens up many new possibilities for biophysical research. Moreover, the size of the cells varied between 2–4 μm in diameter with most of them being close to 3 μm in diameter, which indicates that the average density of the measured cells is around 3 times greater than water density. As expected, the density of the dry mass was significantly higher than the density of water. During measurements water evaporates from the cell while heavy organic structures such as proteins or the cell wall remain in the cell.

It has also been demonstrated that by using our method it is possible to see the change in the mass of a single yeast cell after 6- months of storage. The lyophilized yeast was stored in a fridge as normal in domestic conditions. The moisture was uncontrolled because, as has been mentioned, the yeast were diluted in water. An increase in the mean value of the yeast cell mass was observed. It is a matter of speculation whether yeast cells produced additional proteins during the storage time rather than budding or fully stopping their metabolism.

This work also indicates that the cantilever-based method can be used for the determination of the mass of adherent cells, which is not possible or difficult when using a suspended microchannel method. In addition, specific processes can be detected such as water evaporation and cell positions shift; however, further research is needed.
